# Leptin Gene Polymorphism in Goats Fed with Diet at Different Energy Level: Effects on Feed Intake, Milk Traits, Milk Fatty Acids Composition, and Metabolic State

**DOI:** 10.3390/ani9070424

**Published:** 2019-07-06

**Authors:** Marcella Avondo, Adriana Di Trana, Bernardo Valenti, Andrea Criscione, Salvatore Bordonaro, Anna De Angelis, Daniela Giorgio, Paola Di Gregorio

**Affiliations:** 1 Dipartimento di Agricoltura, Alimentazione e Ambiente (Di3A), University of Catania, Via Valdisavoia 5, 95123 Catania, Italy; 2 Scuola di Scienze Agrarie, Forestali, Ambientali e Alimentari (SAFE), University of Basilicata, Viale dell’Ateneo Lucano 10, 85100 Potenza, Italy; 3 Dipartimento di Scienze Agrarie, Alimentari e Ambientali, University of Perugia, Borgo XX Giugno 74, 06121 Perugia, Italy

**Keywords:** goats, leptin polymorphism, milk yield, blood metabolites, fatty acids

## Abstract

**Simple Summary:**

In this study it has been highlighted the role of a leptin polymorphism on goat milk yield and quality and of its interaction with the energy content of the diet. Energy did not interfere with genotype effect. The studied leptin polymorphism increased healthy fatty acids in milk, thus representing a potential tool to improve functional characteristics of goat milk.

**Abstract:**

The study investigated the effects of a polymorphism at the *LEP* gene intron 1 microsatellite region and its interaction with diet energy level on feed intake, milk traits, milk fatty acid composition, and metabolic state in goats. Sixteen Girgentana lactating goats at mid-lactation, selected on the basis of their genotype (8 goats homozygous 266 bp/266 bp, L genotype; 8 goats heterozygous 266 bp/264 bp, H genotype), were fed ad libitum according to a change-over design, with two diets at different energy levels reached with different hay inclusion: low energy diet (LE)—100% of hay; and high energy diet (HE)—65% of hay. No differences in milk yield and composition or in dry matter intake were found between leptin genotypes or between diets. Leptin genotype had no effect on plasma metabolite concentrations. The differences between diets were recorded for plasma β-hydroxybutyric acid (BHBA) concentrations with higher (*p* = 0.01) values for the HE compared to the LE diet (0.44 vs. 0.24 mmol/L, respectively). Nonesterified fatty acid (NEFA) values seem to indicate a positive energy balance in goats. No interaction genotype per diet was evident for most of the studied parameters. Fatty acid composition was strongly influenced by LEP genotype: L goats, compared to H goats, showed higher levels of monounsaturated fatty acids (MUFA), polyunsaturated fatty acids (PUFA), and 14:1/14:0 desaturation index; lower levels of saturated fatty acids (SFA); and a more favorable atherogenic index. These results seem to suggest an improvement of health characteristics of milk with the L genotype.

## 1. Introduction

Leptin is a protein hormone secreted from white adipose tissue. It may affect food intake, body energy homeostasis, and nutrient partitioning between tissues and plays an important role on energy metabolism. Furthermore, leptin has a regulatory action on lactogenesis [1]. Studies with association analyses revealed the role of leptin gene polymorphisms on the concentration of circulating leptin [2,3] and on the yield and quality traits of meat and milk obtained from farm animal species. In pigs, a single nucleotide polymorphism of the leptin gene was associated with different carcass properties: meatness and average back fat thickness [4]. Moreover, Peixoto et al. [5] reported an effect on the average daily gain, feed conversion, and bacon depth. In sheep, a significant association was found between leptin gene polymorphism and weaning weight [6]. In cattle, leptin or leptin receptor gene polymorphisms have been shown to affect carcass fatty acid composition [7], milk yield [8], milk fat [9,10], milk protein [9], and milk fatty acid composition [11]. Glantz et al. [12] reported an association with milk traits affecting cheese-making, such as gel strength and fat globule size. In goats, Di Gregorio et al. [13] observed, in six different breeds, that two alleles (266 bp and 264 bp) at the *LEP* gene intron 1 microsatellite region were not significantly associated with milk traits, but they were associated with β-hydroxybutyric acid (BHBA), triglycerides, and T4 and IgF1 hormones concentrations in blood. Based on these results, the authors hypothesized that in lactating goats, a different demand for energy is associated with the two alleles. Hernandez et al. [14] found that leptin RNA expression in perirenal adipose tissue of lambs increased linearly with increasing levels of grain in the diet. In light of these findings, the aim of the present study was to evaluate the effects of a leptin gene (*LEP*) intron 1 polymorphism and its interaction with diet energy levels on feed intake, milk yield and composition, and the metabolic state of Girgentana goats.

## 2. Materials and Methods

### 2.1. Animals and Experimental Design

Sixteen Girgentana goats in their 2nd to 4th lactation, homogeneous for milk production (1.1 ± 0.4 kg/day), days of lactation (105 ± 8 days), and body weight (34.9 kg ± 5.2), were selected on the basis of their genotype at the leptin gene (*LEP*) intron 1 microsatellite region as follows: 8 goats homozygous 266 bp/266 bp (L genotype), 8 goats heterozygous 266 bp/264 bp (H genotype). The goats in each genetic group were derived from two different farms. The goats were used according to a change-over design in a 2 × 2 factorial arrangement of treatments, with two genotypes (266 bp/266 bp—L, and 266 bp/264 bp—H) and two diets with different energy levels, reached by varying the hay inclusion in the diets (100% of hay—LE [low energy], and 65% of hay—HE [high energy]). All the animals, managed according to the guidelines of the Animal Ethics Committee of the University of Catania, were housed in individual pens where goats had access to water and salt blocks. The pre-experimental period consisted of a 12-day period during which the animals received a mix of the two experimental diets ad libitum. The experiment lasted 40 days, from 2 March to 11 April. Each experimental period lasted 20 days that’s consisted of 12 days for adaptation and 8 days for data and sample collection during which the goats received the scheduled diet ad libitum.

The animals were fed two different diets with the same protein content but different energy levels: a pelleted alfalfa hay (low energy diet, LE) and a pelleted feed including 65% alfalfa hay (high energy diet, HE) (Table 1). All ingredients were ground and pelleted (6 mm diameter).

### 2.2. Genetic Characterization

Goat DNA samples were obtained from hair bulbs according to Bowling et al. [15]. The primers used for microsatellite genotyping had the following sequences: forward 5′-CCCAGCTCAGGCGACAC-3′ labeled with 6-FAM fluorescent phosphoramidites; and reverse 5′-CCAGGATGCCACAGTGAACA-3′. The PCR reaction was performed in a 20 mL volume containing 50–100 ng DNA; 1× PCR buffer (Promega, Madison, WI, USA); 2.5 mM MgCl_2_; 200 mM of each dNTPs; 0.2 mM of each primer, 1 U Taq DNA polymerase. The PCR conditions consisted of 2 min at 97 °C followed by 30 cycles of 45 s at 94 °C, 45 s at 56 °C, and 45 s at 72 °C. The PCR products were electrophoresed on a 4% denaturing polyacrylamide gel using an ABI PRISM 377 automated DNA sequencer (ABI PRISM, Foster City, CA, USA) and analyzed with GeneScanTM software (ABI) [13].

### 2.3. Sample Collection and Analysis

During the pre-experimental period, every four days, data for milk production and feed intake were measured. During the experimental period, individual intake was measured daily on the basis of refusals. Three samples for each pelleted diet were analyzed for dry matter (DM), crude protein (CP), fat, ash [16], and NDF [17]. Net energy for lactation (NEl) was calculated as reported by Conrad et al. [18]. Individual milk production and milk samples were collected from the morning and evening, milking three times (at 3 days, 5 days, and 8 days) for each 8-day collection period. Milk samples consisting of proportional volumes of morning and evening milk were analyzed for lactose, fat, and protein by an infrared method (Combi-foss 6000, Foss Electric, Hillerød, Denmark). The milk samples collected at the end of each 8-day collection period (8-day samples) were analyzed for fatty acid composition: Milk fat was extracted according to Luna et al. [19] and converted to fatty acid methyl esters (FAME) by base-catalyzed transesterification [20], using 0.5 mL of sodium methoxide in methanol 0.5 N and 1 mL of hexane. Nonadecanoic acid was used as an internal standard. FAME were analyzed on a Trace Thermo Finnigan GC equipped with a flame ionization detector and a 100 m × 0·25 mm i.d. fused-silica capillary column (SP-2560, Supelco, Inc., Bellefonte, PA, USA). Helium was the carrier gas at a constant flow of 1 mL/min. The total FAME profile in a 1 µL sample volume at a split ratio of 1:80 was determined using the GC conditions reported by Valenti et al. [21]. Blood samples (8 mL) were taken from all goats at the end of the pre-experimental period and at the end of each experimental period by jugular venepuncture using vacutainer tubes containing lithium heparin (Becton, Dickinson and Company, Franklin Lakes, NJ, USA) and immediately placed on ice. Within 1 h from the bleeding, blood samples were centrifuged at 1400*g* at 4 °C for 20 min, and plasma was harvested and stored at −20 °C until assayed. A TARGA model 2000 (Technology Advanced Random Generation Analyser, Biotecnica Instruments, Roma, Italy) automated analyzer was used to determine glucose, cholesterol, triglycerides, urea, total protein, and albumin (Mercury, Riardo, Italy) in the plasma samples. Nonesterified fatty acids (NEFA) and beta-hydroxybutyric acid (BHBA) were analyzed using the FA 115 and Ranbut commercial kits (Randox Laboratories, Crumlin, Antrim, UK), respectively.

### 2.4. Statistical Analysis

Individual data for dry matter intake, milk production, and gross composition were analyzed using the GLM procedure for repeated measures in SPSS (SPSS for Windows, SPSS Inc., Chicago, IL, USA). The model included leptin genotype, diets, blocks, periods, and genotype × diet interaction. Pre-experimental data of milk production and dry matter intake (DMI) were used as covariates, respectively, for milk production and composition and for DMI analysis. When a covariate was not significant (*p* > 0.05), it was removed from the model. Milk fatty acids and blood parameters were analyzed using the univariate GLM procedure in SPSS, and analysis included leptin genotype, diet, blocks, and interaction genotype × diet.

## 3. Results

Table 1 shows the ingredients and chemical composition of the two diets. The diets differed markedly in their contents of structural carbohydrates and net energy for lactation.

Table 2 reports the main effects of leptin genotype and diet energy level on dry matter intake, milk yield, and gross composition. No differences in these parameters were evident between leptin genotype and between diets.

In Table 3 blood metabolic parameters are reported. Leptin genotype had no effect on plasma metabolites concentrations. Differences between diets were recorded for plasma β-hydroxybutyric acid (BHBA) concentrations with higher (*p* = 0.01) values for HE compared to LE diet.

Table 4 reports milk fatty acid compositions. Leptin genotype significantly affected some fatty acids: 4:0, 17:0*iso*, 18:0, 18:1 *c*9, 18:1 *c*11, 20:4 n-3, monounsaturated fatty acids (MUFA), polyunsaturated fatty acids (PUFA), and 14:1/14 desaturation index [18] were higher in L goats, whereas 16:0 and saturated fatty acids (SFA) were higher in H goats. Moreover, the atherogenic index, calculated as reported by Ulbricht and Southgate [19], was lower in L goats.

Energy level significantly influenced fatty acid composition: 8:0, 10:0, 12:0, 12:1 *c*9, and 18:2 *c*9*c*12 were higher in goats fed the HE diet, whereas 14:1 *c*9, 15*iso*, 15:0, 17*iso*, 17*anteiso*, 17:0, 17:1 *c*9, 18:1 *c*11, 18:3 *c*9*c*12*c*15, and 14:1/14 desaturation index and the sum of odd and branched chain fatty acids (OBCFA) were higher in goats fed with the LE diet.

## 4. Discussion

The choice of using the goat species to evaluate the effects of leptin gene polymorphism on milk traits and blood parameters lies on the results reported in a previous study [13], where a polymorphism at this gene has been studied on cattle, sheep, goats, and buffaloes. In particular, in the goat species, a significant effect of leptin genotype was found on some metabolic and hormonal parameters, suggesting a different energy utilization in the two identified genotypes. This led us to deepen these results with a feeding trial aimed at evaluating, for the first time, the possible interaction between dietary energy supply and the polymorphism at *LEP* gene intron 1 in a Mediterranean goat breed. The diets in the present study were characterized by different hay inclusions to reach a different energy level with the aim to evaluate whether a leptin polymorphism could act in different ways when energy availability was different.

As reported by Hernandez et al. [14], increasing levels of grain in the diet linearly increased the leptin RNA expression in the perirenal adipose tissue of lambs. However, in our conditions, no interaction between genotype and diet was evident for most of the studied parameters. The polymorphism in the *LEP* gene had no significant effects on dry matter intake, milk yield, and gross composition. These results seem to be in contrast with the studies reported on cattle where several polymorphisms of the leptin gene have been described. These polymorphisms have been associated with significant differences between genotypes in feed intake [8], milk yield [24,25], milk protein [9] and fat [9,10], and the latter is considered the most affected by leptin polymorphisms. To the best of our knowledge, only few studies have investigated the effects of leptin genotypes in lactating goats. Chilliard et al. [26] reviewed that, in goats, lactation per se strongly represses leptin expression whatever the lactation stage. These findings could justify the lack of differences in milk traits found in our experiment between *LEP* genotypes. Surprisingly, no effect of the diet was evident on milk yield and composition. The tendency of goats fed with the HE diet towards higher milk production and lower fat percentage, which represents the typical effect of a diet lower in structural carbohydrates, compared to the LE diet did not reach statistical significance. In a previous study on Girgentana goats, Pagano et al. [27], using experimental diets similar to our diets, found significantly higher milk yield and lower milk fat percentage in goats fed with 65% hay, compared to 100% hay. In our conditions the lack of significance was probably due to high individual variability found for these parameters.

Blood metabolites were not affected by genotype. This is in contrast with the results of Di Gregorio et al. [13], who found that L genotype had higher blood levels of BHBA and triglycerides in Red Syrian goats at early lactation. Moreover, they found lower levels of the thyroid hormone T4 in L goats. Taking into account the positive association of thyroid hormones and the negative association of BHBA and triglycerides with intake and energy balance, the authors argued that these results could demonstrate, for the L genotype, an increased demand for energy. The absence of association between *LEP* genotypes and blood metabolites in Girgentana goats could be explained by the more advanced stage of lactation compared to conditions reported by Di Gregorio et al. [13] or by the population specificity for the effects of the polymorphism. Among the indicators of energy metabolic status (glucose, NEFA, and BHBA), BHBA was the only one affected by the diet. Plasma BHBA originates from two sources: incomplete oxidation of NEFA in the Krebs cycle and metabolism of butyric and acetic acid in the wall of the rumen [28]. In the present experiment, the second source appears to be predominant since BHBA concentrations were higher in goats fed with higher energy levels (HE) compared to goats fed lower energy levels (LE). This result was confirmed by the low levels of NEFA in both groups. In fact, the NEFA concentrations below the critical values (0.20–0.21 mmol/L) [29] suggests that goats did not mobilized body fat reserves and that animals were in the anabolic phase [30].

Fatty acid composition was influenced by *LEP* genotype: L goats, compared to H goats, showed higher levels of 4:0, 17i*so*, 18:0, 18:1 *c*9, 18:1 *c*11, 20:4 n-3, MUFA, and PUFA, and lower levels of 16:0 and SFA. It has been shown that leptin has a strong influence on fatty acids metabolism [31]. The analyzed polymorphism does not seem to be causative of the observed differences that could therefore be explained by the association of polymorphism with a quantitative trait loci (QTL) that influences milk fatty acids composition. In cattle, as in goat, the *LEP* gene maps to chromosome 4, and on the same chromosome, QTLs for fat yield and percentage in milk (Cattle QTL database https://www.animalgenome.org/cgi-in/QTLdb/BT/index) and fatty acid composition are present [32]. In line with results found for the L genotype, Pegolo et al. [11] report that two single nucleotide polymorphisms (SNPs) of the *LEP* gene in dairy cattle were associated with reductions of SFA and increases of MUFA and PUFA. On the contrary, Marchitelli et al. [33] did not find any significant association between a SNP in the *LEP* gene (g. 1180C > T) and the analyzed fatty acid traits in three different bovine breeds. In contrast with the positive energy balance hypothesized for both genotypes, the observed differences found between *LEP* genotypes in fatty acids seem to suggest that the L genotype could be associated with a higher utilization of body fat reserves: In fact, the higher levels of 18:1 *c*9, MUFA, and PUFA, and the lower SFA in this genotype would seem compatible with an increased mobilization of fatty acids from adipose tissue [34,35]. Moreover, this seems to be in accordance with the increased demand for energy hypothesized by Di Gregorio et al. [13] for the L genotype. As reported by Pandit et al. [36], it has been shown that leptin increases energy expenditure through the modulation of blood pressure, heart rate, and the thermogenic capacity of the brown adipose tissue. A higher desaturation activity has been highlighted in the L genotype as demonstrated by the higher 14:1/14 index. Orrù et al. [37] found that three SNPs of the *LEP* gene affect, to different extents, the desaturation of fatty acids into MUFA in muscle fat of Simmenthal bulls. Moreover, they found this leptin polymorphism effect was even higher than the effect of the *SCD1* polymorphism. Cohen et al. [38] found that leptin specifically represses RNA levels and the enzymatic activity of hepatic *SCD1*. The atherogenic index was significantly lower in L goats, thus suggesting a direct or indirect effect of this *LEP* genotype on the improvement of health characteristics of goat milk. As expected, fatty acid composition was affected by diet. On average, the medium chain saturated fatty acids (8:0, 10:0, 12:0) and the 18:2 *c*9*c*12 were higher, whereas the OBCFA (15*iso*, 15:0, 17*iso*, 17*anteiso,* 17:0) and 18:3 *c*9*c*12*c*15 were lower in goats fed the HE diet, which is in line with the concentrate inclusion in their diet, compared to the LE diet. Similar results on fatty acid composition as an effect of a 65% of hay inclusion in the diet, compared to a 100% of hay inclusion, were reported by Valenti et al. [39]. No significant interaction between *LEP* polymorphism and diet was evident in our conditions for fatty acid composition.

## 5. Conclusions

The present study aimed to investigate the role of a leptin intron 1 polymorphism and its interaction with different energy levels of diet on feed intake, milk yield, and quality of Girgentana goats. Results did not highlight any interaction between the studied polymorphism and the experimental diets. However, independently from the diet, the study confirmed the important role of *LEP* polymorphisms on the fatty acid composition of milk. The higher MUFA and PUFA, the lower SFA acids, and the more favorable atherogenic index in the L genotype leads to the hypothesis that there is an improvement of health characteristics of milk in this genotype. If supported by further studies, this result could represent a useful indication for selection.

## Figures and Tables

**Table 1:** Ingredients and chemical composition of the experimental diets.

	LE	HE
*Ingredients %*		
Pelleted alfalfa hay	98.0	65.0
Maize meal	-	16.0
Barley meal	-	8.0
Soybean meal	-	3.0
7Carob pulp	-	3.0
Corn gluten meal	-	3.0
Vitamin-mineral premix	2.0	2.0
*Chemical composition*		
Dry matter %	89.0	87.0
Crude protein % dry matter (DM)	15.7	15.8
Neutral detergent fiber % DM	55.2	45.0
Lignin % DM	12.8	7.0
Crude lipids % DM	1.8	2.5
Ash % DM	8.8	8.2
NEl^1^ kcal/kg DM	1151.1	1404.1

LE, low energy level; HE, high energy level; ^1^ Nel, net energy for lactation [18].

**Table 2:** Effects of leptin genotype and energy level of diet on milk yield and composition and dry matter intake.

Goat Production Parameters and Intake	Leptin Genotype (G)		Diet Energy Level (E)		*p-*Value			SEM
	H	L	LE	HE	G	E	G ^×^ E	
Milk yield g/day	1053.7	974.2	891.9	1136.0	0.637	0.156	0.823	69.4
Fat %	3.23	3.27	3.45	3.06	0.836	0.057	0.482	0.10
Protein %	3.45	3.70	3.60	3.55	0.127	0.778	0.642	0.07
Lactose %	4.29	4.32	4.29	4.31	0.709	0.837	0.942	0.04
Dry matter intake g/day	2529.3	2518.6	2519.2	2528.7	0.532	0.577	0.250	10.1

H, L genotype corresponded to 266 bp/264 bp and 266 bp/266 bp phenotype, respectively; LE, low energy level; HE, high energy level.

**Table 3:** Effect of leptin genotype and energy level of diet on blood metabolites.

	Leptin Genotype (G)		Diet Energy Level (E)		*p*-Value			SEM
	H	L	LE	HE	G	E	G ^×^ E	
Glucose (mg/100 mL)	47.1	48.3	48.6	46.9	0.707	0.576	0.877	1.17
NEFA^1^ (mmol/L)	0.12	0.14	0.16.	0.11.	0.562	0.206	0.348	0.02
BHBA^2^ (mmol/L)	0.35	0.33	0.24 ^a^	0.44 ^b^	0.770	0.010	0.675	0.03
Cholesterol (mg/100 mL)	65.2	60.8	60.6	66.4	0.423	0.429	0.502	2.37
Triglycerides (mg/100 mL)	12.0	12.1	11.7	12.4	0.938	0.735	0.766	0.95
Urea (mg/100 mL)	51.4	50.8	53.3	48.9	0.885	0.260	0.714	1.70
Total protein (g/100 mL)	7.1	7.5	7.5	7.1	0.298	0.226	0.738	0.15
Albumin (g/L)	26.6	27.1	27.0	26.7	0.162	0.491	0.477	0.21
Globulin (g/L)	44.7	48.1	48.2	44.2	0.378	0.264	0.693	1.54
Calcium (mg/100 mL)	7.94	8.03	8.16	7.81	0.830	0.360	0.725	0.14
Phosphorus (mg/100 mL)	3.7	3.8	3.7	3.9	0.860	0.482	0.955	0.10
Magnesium (mg/100 mL)	3.0	3.5	3.2	3.3	0.052	0.517	0.224	0.09

H, L genotype corresponded to 266 bp/264 bp and 266 bp/266 bp phenotype, respectively; LE, low energy level; HE, high energy level. ^1^ NEFA, non-esterified fatty acids. ^2^ BHBA, β-hydroxybutyrate. ^a,b^ Values within a row with different superscripts differ significantly at *p* ≤ 0.05.

**Table 4:** Effects of leptin genotype and energy level of diet on milk fatty acid composition.

Fatty Acid Chain Size Examined	Leptin Genotype (G)		Diet Energy Level (E)		*p*-Value			SEM
	H	L	LE	HE	G	E	G ^×^ E	
4:0	2.4 ^a^	2.7 ^b^	2.5	2.6	0.005	0.141	0.110	0.06
6:0	2.7	2.7	2.6	2.8	0.748	0.152	0.545	0.05
8:0	2.8	2.9	2.7 ^a^	3.0 ^b^	0.516	0.017	0.210	0.07
9:0	0.12	0.13	0.14	0.12	0.337	0.238	0.175	0.01
10:0	11.4	11.2	10.5 ^a^	12.2 ^b^	0.634	<0.001	0.017	0.24
11:0	0.25	0.23	0.22	0.27	0.417	0.064	0.559	0.01
12:0	6.2	6.4	5.8 ^a^	6.9 ^b^	0.545	0.006	0.054	0.20
12:1 *c*9	0.21	0.23	0.19 ^a^	0.25 ^b^	0.433	0.038	0.230	0.01
13:0	0.11	0.13	0.13	0.11	0.089	0.051	0.208	0.01
14:0	13.7	12.7	12.7	13.7	0.089	0.076	0.441	0.24
14:1 *c*9	0.10	0.12	0.15 ^a^	0.08 ^b^	0.133	<0.001	0.222	0.01
15*iso*	0.22	0.26	0.30 ^a^	0.19 ^b^	0.123	0.001	0.634	0.02
15*anteiso*	0.23	0.21	0.22	0.22	0.340	0.962	0.508	0.01
15:0	1.1	1.2	1.5 ^a^	0.8 ^b^	0.107	<0.001	0.734	0.08
16:0	29.0 ^a^	24.7 ^b^	27.3	26.3	<0.001	0.314	0.181	0.57
17*iso*	0.30 ^a^	0.36 ^b^	0.39 ^a^	0.26 ^b^	0.022	<0.001	0.144	0.02
16:1 *c*9	0.69	0.63	0.71	0.61	0.243	0.058	0.130	0.02
17*anteiso*	0.41	0.49	0.54 ^a^	0.36 ^b^	0.069	0.001	0.431	0.03
17:0	0.73	0.73	0.93 ^a^	0.57 ^b^	0.440	<0.001	0.934	0.05
17:1 *c*9	0.30	0.33	0.40 ^a^	0.23 ^b^	0.417	0.001	0.193	0.02
18:0	5.4 ^a^	6.7 ^b^	6.5	5.6	0.019	0.104	0.213	0.24
18:1 *t*9	0.20	0.22	0.21	0.21	0.452	0.857	0.671	0.01
18:1 *t*11	0.11	0.11	0.12	0.10	0.935	0.251	0.458	0.01
18:1 *c*9	13.3 ^a^	15.3 ^b^	14.8	13.7	0.009	0.134	0.198	0.38
18:1 *c*11	0.36 ^a^	0.44 ^b^	0.45 ^a^	0.35 ^b^	0.002	<0.001	0.088	0.02
18:2 *c*9*c*12	4.0	4.7	3.6 ^a^	5.1 ^b^	0.110	0.001	0.063	0.25
18:3 *c*9*c*12*c*15	1.5	1.8	2.1 ^a^	1.1 ^b^	0.150	<0.001	0.914	0.16
18:2 *c*9*t*11	1.1	1.2	1.1	1.1	0.434	0.668	0.528	0.05
*18:2 t*10*c*12	0.07	0.11	0.07	0.10	0.184	0.284	0.515	0.01
20:4 n-3	0.34 ^a^	0.42 ^b^	0.39	0.37	0.030	0.548	0.476	0.02
Σ SFA	76.3 ^a^	73.8 ^b^	75.1	75.2	0.050	0.993	0.322	0.48
Σ MUFA	15.0 ^a^	17.3 ^b^	16.9	15.4	0.009	0.068	0.151	0.43
Σ PUFA	7.1 ^a^	8.2 ^b^	7.3	7.9	0.030	0.278	0.153	0.24
Σ *trans* total	0.55	0.56	0.57	0.54	0.874	0.661	0.714	0.03
14:1/14 ^1^	0.75 ^a^	0.99 ^b^	1.18 ^a^	0.56 ^b^	0.042	<0.001	0.107	0.09
AI ^2^	4.1 ^a^	3.3 ^b^	3.6 ^a^	3.9 ^b^	0.006	0.205	0.882	0.13

H, L genotype corresponded to 266 bp/264 bp and 266 bp/266 bp phenotype, respectively. LE, low energy level; HE, high energy level. SFA, saturated fatty acids; MUFA, monounsaturated fatty acids; PUFA, polyunsaturated fatty acids. ^1^ 14:1/14 desaturation index [22]. ^2^ Atherogenic index [23]. ^a,b^ Values within a row with different superscripts differ significantly at *p* ≤ 0.05.
